# EphrinA4 mimetic peptide impairs fear conditioning memory reconsolidation in lateral amygdala

**DOI:** 10.1038/s41598-022-21519-3

**Published:** 2022-10-22

**Authors:** Ron Mana, Or Ilovich, Monica Dines, Raphael Lamprecht

**Affiliations:** Sagol Department of Neurobiology, Faculty of Natural Sciences, University of Haifa, Haifa, Israel

**Keywords:** Amygdala, Classical conditioning, Fear conditioning, Long-term memory

## Abstract

Fear memory may undergo a process after memory reactivation called reconsolidation. To examine the roles of ephrinA4 in fear memory reconsolidation an inhibitory ephrinA4 mimetic peptide (pep-ephrinA4), that targets the EphA binding site and inhibits EphA activation, was used. Pep-ephrinA4 was microinjected into the lateral amygdala (LA) of fear-conditioned rats 24 h after training and 30 min before tone CS memory retrieval. Memory retrieval was unaffected by pep-ephrinA4. However, the animals were impaired in fear memory tested 1 h or 24 h afterward when compared to controls. Fear-conditioned animals injected with pep-ephrinA4 into LA immediately after long-term memory retrieval were unaffected when tested 24 h afterward. Microinjection into LA of a peptide originated from an ephrinA4 site that does not interact with EphA did not affect fear memory reconsolidation. Rats that were administrated with pep-ephrinA4 systemically 24 h after fear conditioning and 30 min before CS memory retrieval were impaired in long-term fear conditioning memory tested 24 h afterward when compared to the control peptide. These results show that ephrinA4 binding sites are needed for long-term fear memory reconsolidation in LA and may serve as a target for the treatment of fear-related disorders by blocking reconsolidation.

## Introduction

Memories become stable after learning in the brain during the consolidation process where cellular and molecular events lead to the establishment of the memory trace^1–4^. However, it has been shown that a consolidated memory needs to be reconsolidated following its reactivation to become stable again even long after the initial learning^5–9^. The opportunity to modify memory in the process of reconsolidation holds great clinical potential as it enables to target memories that contribute to pathological conditioning^10^, such as fearful memories, long after they occurred. However, in some behavioral paradigms older memories are more resistant to interference with time^11^.

In this study, we aimed to explore molecular mechanisms of fear memory reconsolidation. We focused on the roles of EphA receptors and ephrinA in fear memory reconsolidation. EphA receptors and ephrinA are involved in the regulation of neuronal morphology and synaptic transmission during development and in the adult brain^12–14^. Memory reconsolidation involves neuronal transmission and neuronal morphogenesis^15,16^. We were therefore interested to explore the possibility that EphA and ephrinA are needed for fear memory reconsolidation. Specifically, we study the roles of ephrinA4 in fear conditioning memory reconsolidation. EphrinA4 is involved in the regulation of neuronal morphogenesis^17^. Furthermore, EphA4, which has a very high affinity to ephrinA4^18^, is involved in synaptic plasticity in the amygdala, as long-term potentiation (LTP) induced in amygdala synapses is impaired in EphA4^−/−^ mice^19^. To study ephrinA4 roles in fear memory in amygdala, we designed an EphA binding site targeted inhibitory ephrinA4 mimetic peptide (pep-ephrinA4). We showed in a previous study that this ephrinA4 peptide binds EphA4, inhibits EphA4 activation by ephrinA4 and impairs fear memory formation in LA^20^.

Fear conditioning is one of the most straightforward behavioral paradigm that is used to study fear-related disorders^21,22^. In this paradigm, the animal associates a neutral stimulus, (e.g. a tone) with an aversive stimulus, such as mild footshock^23–26^. Fear conditioning is useful for studying the molecular mechanisms underlying fear memory as the site essential for fear memory formation, the lateral nucleus of the amygdala (LA), has been discovered^27,28^. The LA is also needed for fear memory reconsolidation. For example, inhibition of protein synthesis in LA disrupted memory reconsolidation^5^. Thus, fear conditioning can be used to assess cellular and molecular mechanisms that might mediate long-term fear memory reconsolidation and to further explore therapeutically pharmacological approaches for the treatment of fear-related disorders, such as posttraumatic stress disorder and phobias through interfering with the reconsolidation process. We, therefore, study the effects of the pep-ephrinA4 in LA on fear memory reconsolidation.

## Results

### Injection of ephrinA4 mimetic peptide into the LA before memory retrieval impaired memory reconsolidation

**Figure 1:**
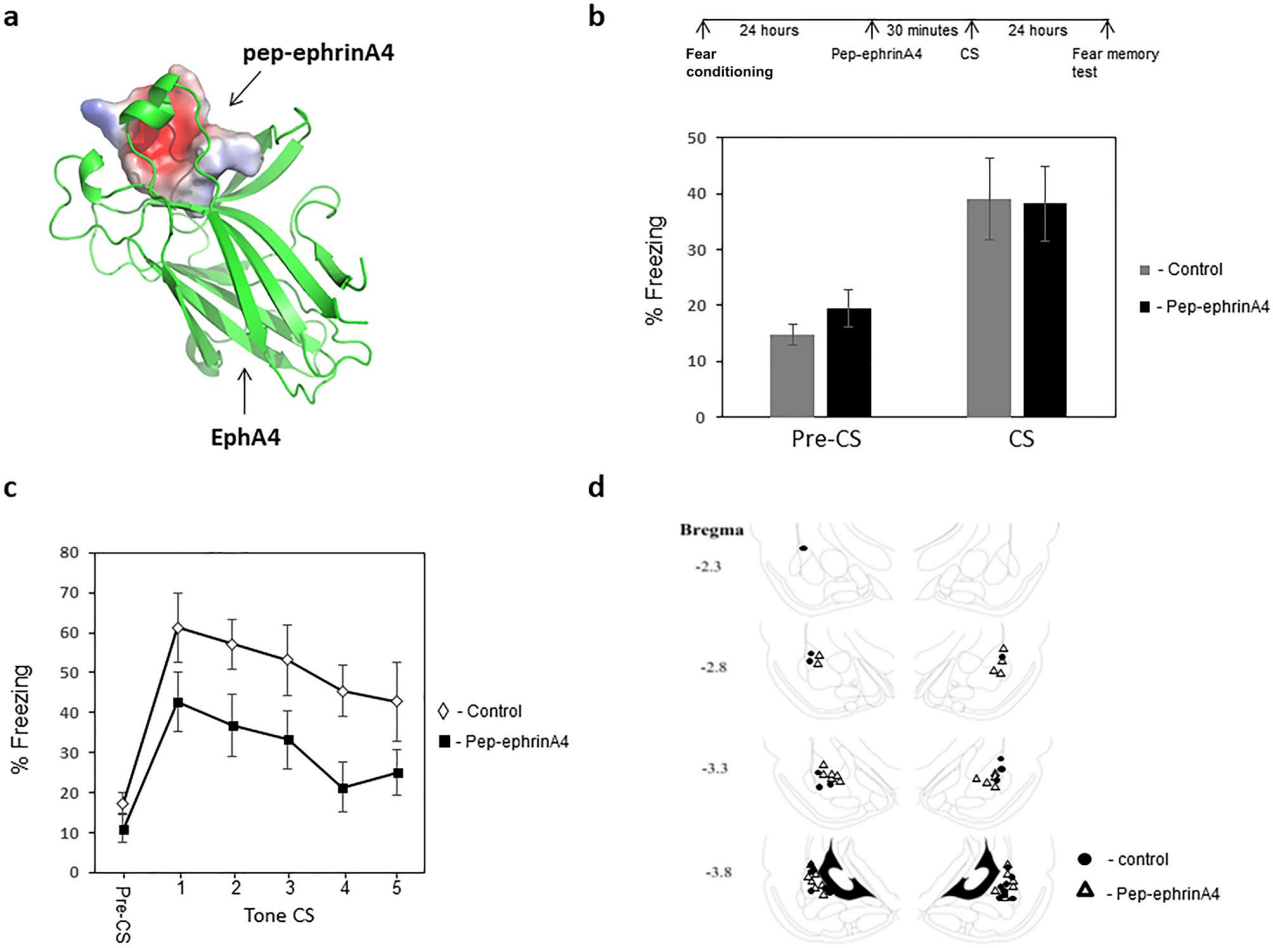
Injection of ephrinA4 mimetic peptide into the LA before memory retrieval impaired memory reconsolidation. **(a)** A molecular model of pep-ephrinA4 peptide bound to EphA4. **(b)** Rats were trained for fear conditioning and 24 h afterward injected with pep-ephrinA4 30 min before fear memory reactivation (CS presentation) and the effect on fear memory was studied a day later. The pep-ephrinA4 (n = 15) and control solution (n = 12) microinjected rats were not significantly different in freezing response to the CS during memory reactivation (p = 0.792). **(c)** Microinjection of pep-ephrinA4 30 min before fear memory retrieval impaired long-term fear memory tested 24 h afterward compared to control injected rats (F_(1,25)_ = 5.473, p = 0.028). **(d)** Description of cannula tip placements.

To study the roles of ephrinA4 in fear memory reconsolidation an ephrinA4 peptide (pep-ephrinA4) derived from the ephrinA4 binding domain (GH loop) was designed according to the structure provided by a molecular model (Fig. 1a)^20^. Pep-ephrinA4 binds to EphA4 and inhibits EphA4 phosphorylation induced by ephrinA4^20^.

We were interested to explore the possibility that ephrinA4 interaction with EphA is needed for reconsolidation of fear memory. Toward that end, rats were subjected to a single tone (conditioned stimulus, CS) that co-terminated with a foot-shock (unconditioned stimulus, US). Twenty-four hours after training the rats received a bilateral infusion of pep-ephrinA4 or control solution into the LA (Fig. 1d). We used a similar concentration of the pep-ephrinA4 that was found to be useful to inhibit ephrinA4 activation of EphA^20^. Thirty minutes after infusion the rats were subjected to a single CS presentation (Fig. 1b). The pep-ephrinA4 (n = 15) and control solution (n = 12) microinjected rats were not significantly different in freezing response to the CS during memory reactivation (p = 0.792) (Fig. 1b). Microinjection of pep-ephrinA4 30 min before retrieval impaired long-term fear memory tested 24 h afterward when compared to rats injected with the control solution that showed normal freezing levels (F_(1,25)_ = 5.473, p = 0.028) (Fig. 1c). The group × tone trial interaction did not differ significantly (F_(2.835,70.887)_ = 0.124, p = 0.939). The above results show that ephrinA4 binding sites in LA during memory retrieval are essential for memory reconsolidation.

### Injection of pep-ephrinA4 into LA before memory retrieval impairs fear memory tested an hour after memory reactivation

**Figure 2:**
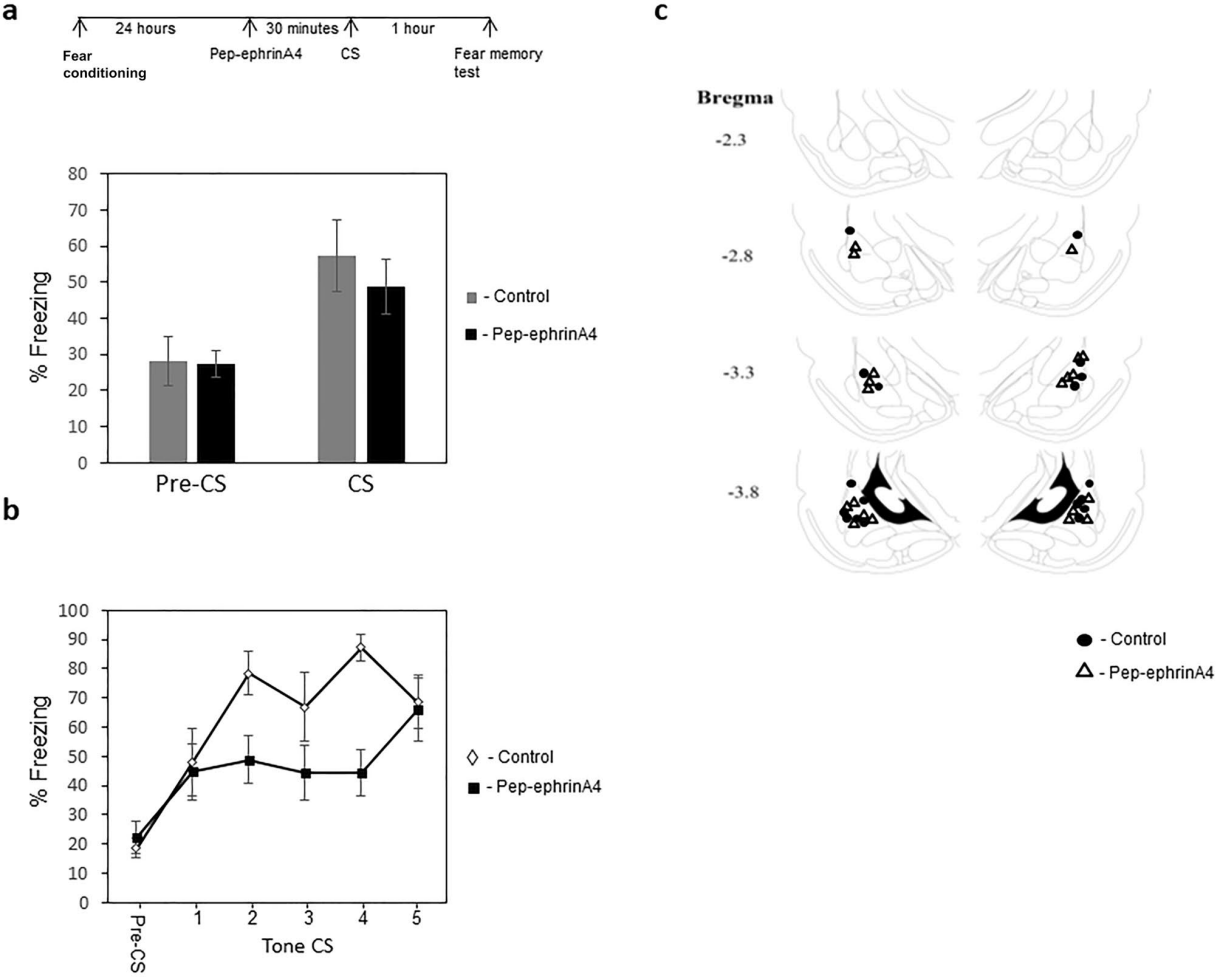
Injection of pep-ephrinA4 before fear conditioning impairs fear memory tested 1 h after memory reactivation. **(a)** Rats were trained for fear conditioning and 24 h afterward injected with pep-ephrinA4 30 min before fear memory reactivation (CS presentation) and the effect on fear memory was studied an hour later. The pep-ephrinA4 (n = 10) and control solution (n = 9) microinjected rats were not significantly different in freezing response to the CS during memory reactivation (p = 0.518). **(b)** Microinjection of pep-ephrinA4 30 min before fear memory retrieval impaired fear memory tested 1 h afterward compared to control injected rats (F_(1,17)_ = 8.921 p = 0.008). **(c)** Description of cannula tip placements.

To examine the temporal effect of pep-ephrinA4 on memory reconsolidation we injected it into the LA 30 min before memory retrieval and examined fear conditioning memory 1 h after memory reactivation. We observed that injection of the pep-ephrinA4 has no effect on memory retrieval (Fig. 2a). The pep-ephrinA4 injected animals (n = 10) are not significantly different from the control solution injected rats (n = 12) (p = 0.518). The animals injected with pep-ephrinA4 were impaired in fear memory compared to control solution-injected rats when tested 1 h after reactivation (F_(1,17)_ = 8.921 p = 0.008) (Fig. 2b). The group × tone trial interaction did not differ significantly (F_(2.61, 44.377)_ = 1.897, p = 0.151). Thus, pep-ephrinA4 has no effect on memory retrieval but affects fear memory tested 1 h after memory reactivation.

### Injection of ephrinA4 mimetic peptide into the LA immediately after memory retrieval does not affect memory reconsolidation

**Figure 3:**
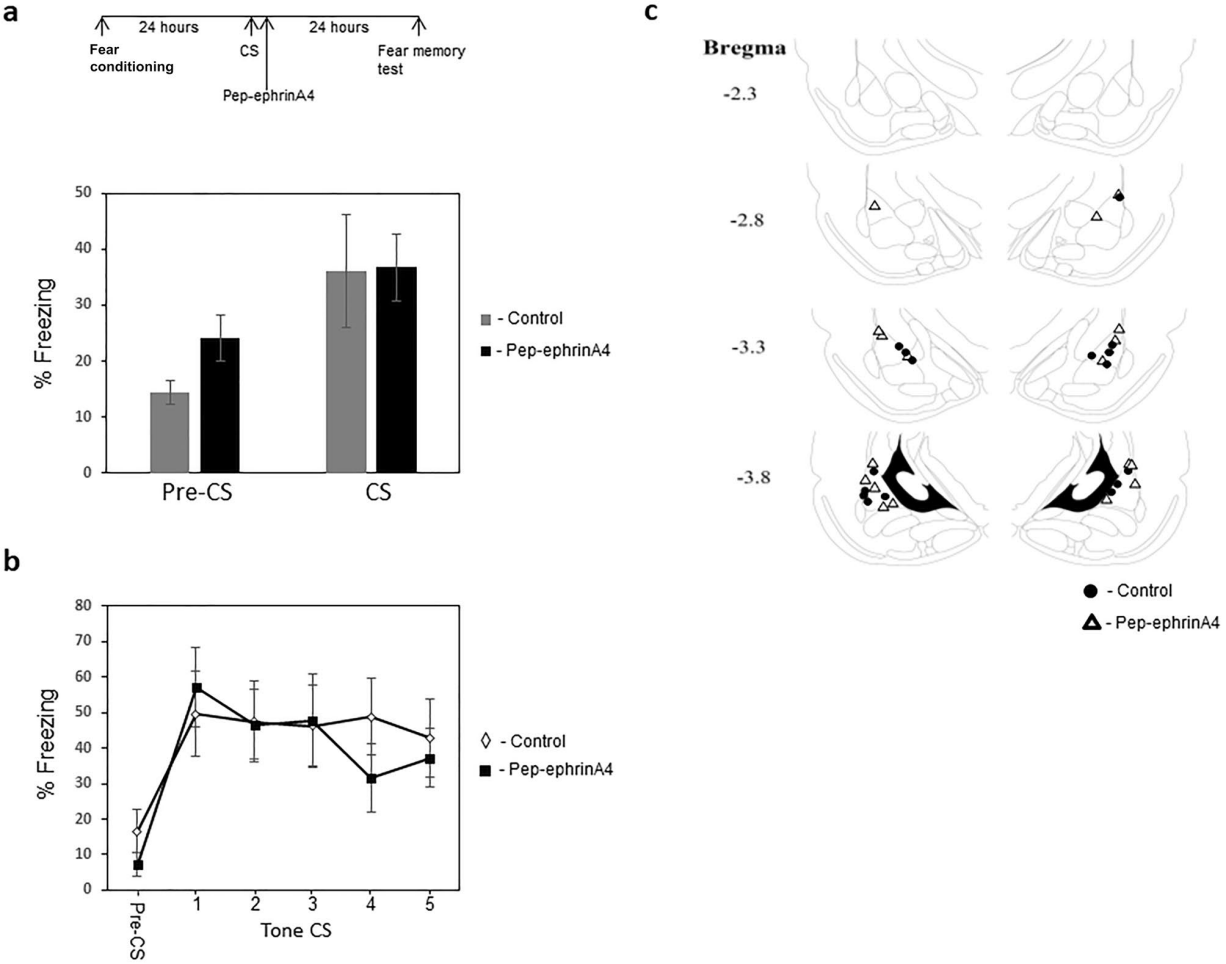
Injection of ephrinA4 mimetic peptide into the LA immediately after memory retrieval does not affect memory reconsolidation. **(a)** Rats were fear conditioned and twenty-four hours later received a single CS presentation that followed immediately afterward with bilateral infusions of pep-ephrinA4 or control solution into the LA. Fear memory is tested 24 h afterward. The pep-ephrinA4 (n = 9) and control solution (n = 8) microinjected rats were not significantly different in freezing response to the CS during memory reactivation (p = 0.95). **(b)** There is no significant difference in long-term memory between the peptide or control solution injected animals F_(1,15)_ = 0.049, p = 0.827) 24 h after memory reactivation showing that injection of pep-ephrinA4 into LA after fear memory reactivation does not affect memory reconsolidation. **(c)** Description of cannula tip placements.

The above results show that inhibition of ephrinA4 interaction with its EphA receptor target before fear memory retrieval impaired memory reconsolidation. We examined whether ephrinA4/EphA interaction is needed also post reactivation for memory reconsolidation. To explore this possibility, we injected pep-ephrinA4 into LA (Fig. 3c) immediately after fear memory retrieval and studied the effect on memory reconsolidation (Fig. 3a). The pep-ephrinA4 (n = 9) and control solution (n = 8) microinjected rats were not significantly different in freezing response to the CS during memory reactivation (p = 0.95) (Fig. 3a). There is no significant difference in long-term memory between the pep-ephrinA4 peptide or control solution injected animals 24 h after memory reactivation (F_(1,15)_ = 0.049, p = 0.827) (Fig. 3b). Both groups show normal freezing levels. The group × tone trial interaction did not differ significantly (F_(2.627, 39.405)_ = 0.992, p = 0.398), indicating that pep-ephrinA4 did not alter freezing over the trials when compared with control. These results show that injection of pep-ephrinA4 into LA after fear memory reactivation does not affect memory reconsolidation.

### Injection of a control inactive peptide from a non-binding site of ephrinA4 does not affect memory reconsolidation

**Figure 4:**
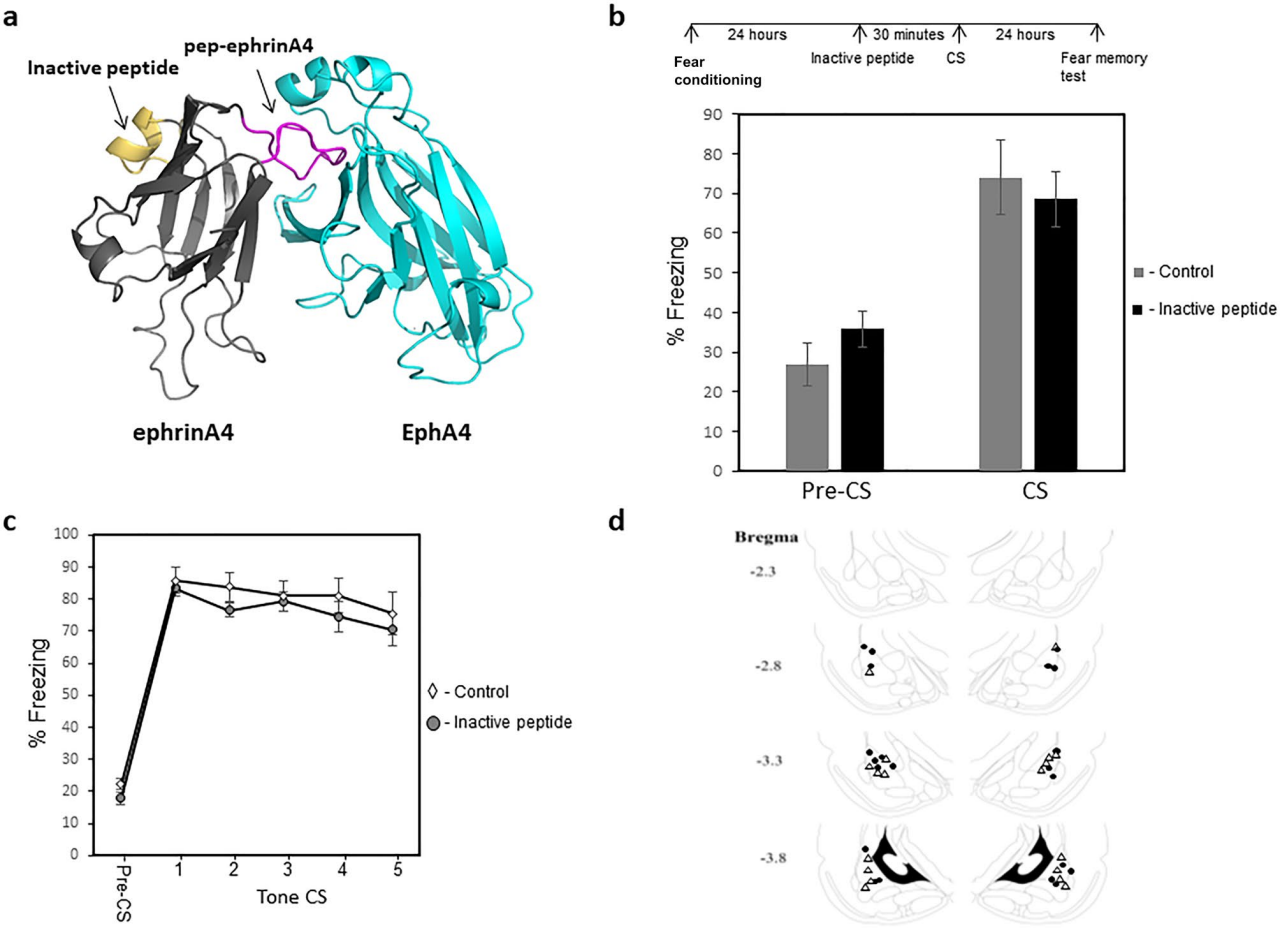
Injection of a control inactive peptide into the LA does not affect fear memory reconsolidation. **(a)** The inactive peptide is derived from a non-binding site of ephrinA4 (yellow). **(b)** The inactive peptide did not affect freezing during the presentation of the CS memory reactivation (p = 0.21). **(c)** There is no significant difference in long-term memory between the inactive peptide (n = 11) and control solution (n = 9) injected animals 24 h after memory reactivation (F_(1,18)_ = 0.728, p = 0.405). **(d)** Cannula tip placements.

The results above show that pep-ephrinA4 injection before CS memory reactivation impaired long-term memory reconsolidation. To test whether this is a nonspecific effect of the pep-ephrinA4 we injected a peptide comprised of a nonbinding E helix site of ephrinA4 (Fig. 4a) into the amygdala (Fig. 4d) 30 min before CS memory reactivation (Fig. 4b). The inactive control peptide had no effect on freezing during the presentation of the CS memory reactivation (p = 0.21) (Fig. 4b). There is no significant difference in long-term memory between the inactive peptide (n = 11) and control solution (n = 9) injected animals 24 h after memory reactivation (F_(1,18)_ = 0.728, p = 0.405) (Fig. 4c). The group × tone trial interaction did not differ significantly (F_(2.532, 45.572)_ = 0.593, p = 0.595), indicating that the inactive peptide did not alter freezing over the trials when compared with the control. These results show that injection of the inactive peptide into LA before fear memory reactivation does not affect memory reconsolidation. Cumulatively, these results show that the effect of pep-ephrinA4 is specific and that the ephrinA4 binding site is needed for memory reconsolidation.

### Acute systemic injection of pep-ephrinA4 before memory retrieval impaired memory reconsolidation

**Figure 5:**
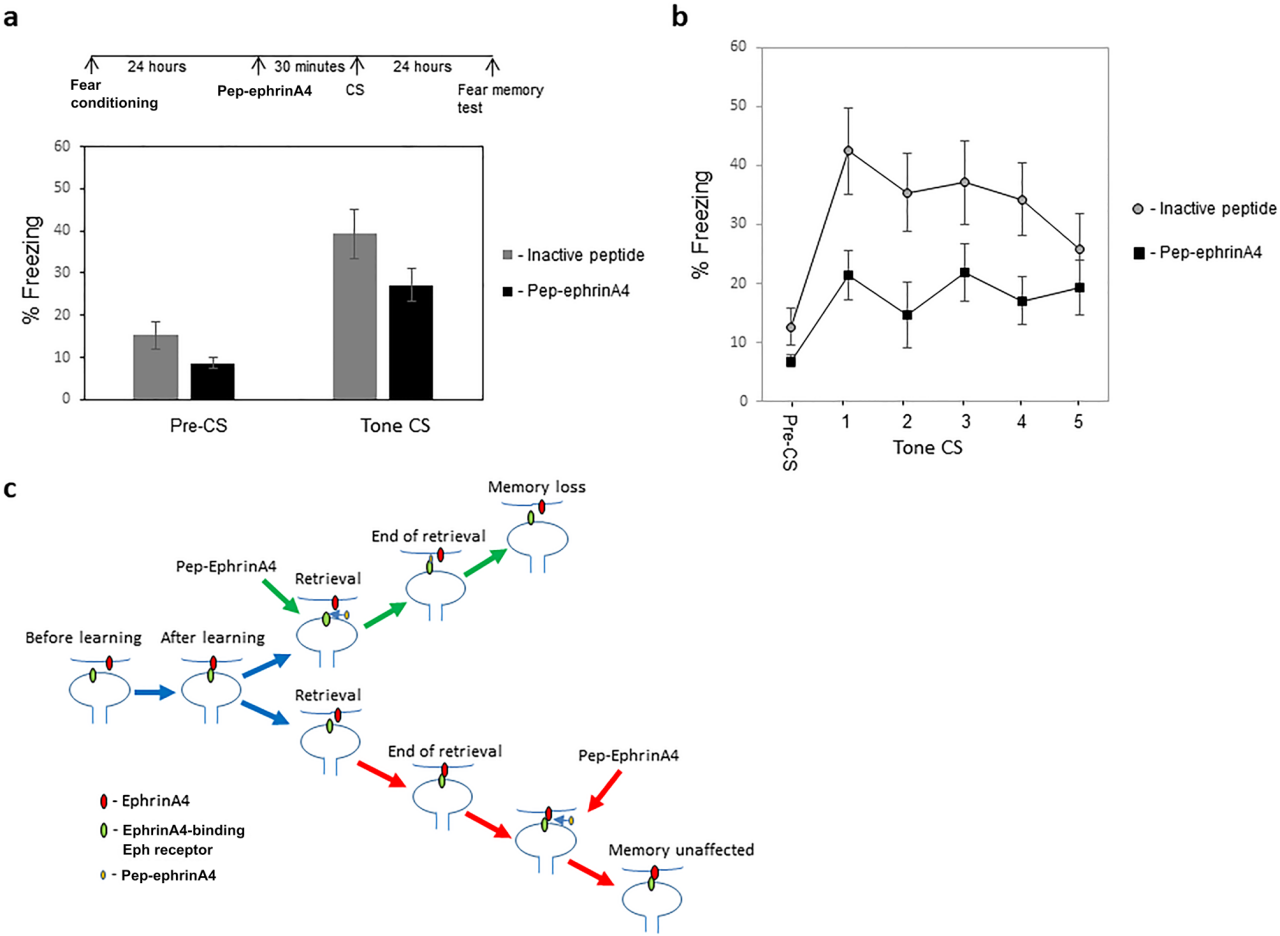
Acute systemic injection of pep-ephrinA4 before memory retrieval impaired memory reconsolidation. **(a)** Injection of the peptide (n = 18) before memory reactivation did not lead to a significant change in response to the tone CS when compared to injection of the inactive peptide (n = 18) (p = 0.09). **(b)** Rats that were injected with the pep-ephrinA4 30 min before fear memory retrieval were significantly impaired in fear memory tested 24 h afterward compared to animals injected with the inactive peptide (n = 18) (F_(1,34)_ = 5.812, p = 0.021). **(c)** A model for a role for ephrinA4 and EphA in fear memory reconsolidation. Fear conditioning training leads to the binding of EphA to ephrinA. During auditory memory reactivation synaptic ephrinA4 briefly dislodges from EphA allowing for molecular intervention. This is followed by appropriate re-connections of EphA and ephrinA for auditory fear conditioning memory to reconsolidate. However, if these interactions are disrupted (e.g. by the pep-ephrinA4) fear memory cannot reconsolidate. This is the reason why pre-reactivation injection (green pathway) of the pep-ephrinA4 affects reconsolidation whereas post-reactivation injection (red pathway) at the time when the EphA and ephrinA are already re-bound has no effect. The peptide needs to be at the synapse during memory reactivation to affect the binding of ephrinA to EphA (green pathway).

The aforementioned results show that ephrinA4 binding sites in LA may serve as a target for pharmacological treatment of fear-related disorders by injecting it before reconsolidation of the fearful memory. For future drug development, it would be useful to test the pep-ephrinA4 effect on memory reconsolidation by applying it systemically. To test such an effect, we trained the animals for fear conditioning and a day later subjected them to acute subcutaneous injection of pep-ephrinA4 30 min before CS presentation to examine the effect of the peptide on fear memory tested 24 h afterward (Fig. 5a). As a control, we used the inactive peptide derived from an area that does not bind EphA receptors. Injection of the pep-ephrinA4 (n = 18) before the CS memory reactivation did not lead to a significant difference in response to the tone CS when compared to injection of the control inactive peptide (n = 18) (p = 0.09) (Fig. 5a). Rats injected with the pep-ephrinA4 30 min before fear memory retrieval were significantly impaired in fear memory when tested 24 h later compared to animals injected with the inactive peptide (F_(1,34)_ = 5.812, p = 0.021) (Fig. 5b). The treatment × tone trial interaction was not significant (F_(3.015,102.517)_ = 1.334, p = 0.267). These results show that systemic administration of the pep-ephrinA4 before fear memory retrieval impairs long-term fear memory reconsolidation.

## Discussion

EphrinAs and their cognate EphA receptors are key proteins intimately involved in regulating synaptic transmission and morphogenesis during brain development and in adults^12–14^. We were therefore interested to explore the possibility that ephrinA4 and EphA receptors are involved in fear memory reconsolidation. The present study shows that microinjection of an inhibitory ephrinA4 mimetic peptide, targeted to EphA binding sites, into LA 30 min before but not after fear memory reactivation impaired the reconsolidation of auditory fear conditioning memory. Injection of a control inactive peptide from a nonbinding site of ephrinA4 has no effect. Furthermore, acute subcutaneous injection of pep-ephrinA4 30 min before fear memory reactivation impaired the ability to reconsolidate fear memory. We do see that the subcutaneous injection of the pep-ephrinA4 leads to a reduction, although not significant, of the freezing during reactivation. However, the end result is similar and thus pep-ephrinA4 can potentially be used as a drug during memory reactivation treatment. These results indicate that ephrinA4 binding sites are needed for fear memory reconsolidation and that they may serve as potential targets for therapeutically pharmacological intervention in fear-related disorders.

We observed that microinjection of pep-ephrinA4 into LA impaired fear memory reconsolidation when injected before fear memory reactivation but not after reactivation. These results suggest a mechanism whereby ephrinA4 and its cognate EphA receptor are involved in reconsolidation (Fig. 5c). During fear conditioning EphA receptors and ephrinAs make contacts important for memory consolidation. During auditory memory reactivation, ephrinA4 in LA is disconnected briefly and opens a short time window for intervention. After reconsolidation ephrinA4 reconnects with its cognate EphA receptor. If appropriate connections are made auditory fear conditioning memory reconsolidates. However, if these interactions are disrupted fear memory cannot reconsolidate. This is the reason why pre-reactivation injection of the pep-ephrinA4 affects reconsolidation whereas post-reactivation does not affect it. The peptide needs to be at the synapse during memory reactivation to impair the binding of ephrinA4 to EphA. The binding of ephrinA4 to EphA is completed rapidly during reactivation as post-reactivation injection of pep-ephrinA4 has no effect. In our previous study^20^ we have shown that post-subcutaneous injection of pep-ephrinA4 affects long-term memory. It could be that the period when ephrinA and EphA are still open is longer after fear conditioning learning (in amygdala or other brain regions) than after reactivation and therefore is still sensitive post training to pep-ephrinA4 injection.

It has been shown that NMDA receptors blockade in LA before reactivation blocks the initiation of reconsolidation (destabilization), as it leads to the reactivated memory being insensitive to subsequent reduction by protein synthesis inhibitors^29^. Post memory reactivation infusion of NMDA receptor antagonist (during restabilization of memory) does not affect the ability of protein synthesis inhibitor to impair memory reconsolidation. Thus, NMDA receptors in the amygdala are needed for transforming memory into a labile state. It would be interesting to study whether activation of NMDA receptor during reactivation affects EphA receptor or vice versa.

It would be also interesting to explore how EphA4 that binds to ephrinA4 affects memory consolidation and reconsolidation. EphA4 signaling can affect actin-regulatory proteins (e.g. cofilin^30^). Actin cytoskeleton polymerization is needed for fear conditioning long-term memory consolidation and reconsolidation in BLA^31,32^. Since ephrinA4 in LA affects both consolidation^20^ and reconsolidation the results suggest that it may exert its effect through EphA regulation of actin cytoskeleton.

Interestingly, EphA4 signaling is a critical mechanism for astrocytes to regulate synaptic function and plasticity. Astrocytes receive a signal from postsynaptic EphA4 receptors via ephrinA, which prevents them from upregulating glial glutamate transporter expression to high levels and thereby control glutamate concentrations near synapses and promote LTP^33–35^. EphrinA4 is expressed in astrocytes^36^ and it therefore will be intriguing to explore whether ephrinA4 in astrocytes may exert its effects on fear memory formation and reconsolidation.

We show here that injection of pep-ephrinA4 into LA affects memory shortly after reactivation. This indicates that the effect of the peptide is rapid and that both STM and LTM after reactivation require proper binding of the EphA and ephrinA. Interestingly, in our previous study, we found that pre fear conditioning injection of pep-ephrinA4 has an effect of LTM but not on STM suggesting that ephrinA and EphA have a different role in consolidation and reconsolidation events.

It would be interesting also to explore pep-ephrinA4 effects on STM and LTM in other amygdala nuclei, such as the central nucleus of the amygdala, that are also involved in fear memory reconsolidation^37^.

In summary, we show that pep-ephrinA4 injection directly into the LA or systemically before reactivation impairs memory reconsolidation. This observation indicates that this peptide could serve as a therapeutic drug to treat diseases associated with traumatic memories such as post-traumatic stress disorder (PTSD) by injecting it systemically before fearful memory reactivation.

## Materials and methods

### Animals

Male Sprague–Dawley rats (225–250 g) were used in the study (Harlan Laboratories, Jerusalem, Israel). Following surgery, the rats were housed separately at 22 ± 2 °C in a 12 h light/dark cycle, with free access to food and water. Behavioral experiments were approved by the University of Haifa Institutional Committee for animal experiments in accordance with National Institutes of Health guidelines. All animal studies described were performed in concordance with the ARRIVE guidelines.

### Surgical procedures

Rats were anesthetized with xylazine 2% (15 mg/kg) and ketamine 100 mg/ml (120 mg/kg). Calmagine (Vetoquinol) (0.01 ml) was injected for analgesia before surgery. Guide stainless-steel cannulas (23 gauge) were implanted bilaterally 1.5 mm above the LA (LA coordinates are in reference to bregma: anteroposterior (AP), − 3.0; lateral (L) ± 5.2; and dorsoventral (DV), − 8.0). Following surgery, the rats received antibiotics (0.25 ml; Pen and Strep, Norbrook, Newry, Northern Ireland). The animals recovered for 5–7 days before behavioral training^20^.

### Microinjection

The stylus was removed from the guide cannula and a 28-gauge injection cannula, extending 1.5 mm from the tip of the guide cannula aimed to the LA was carefully placed. The injection cannula was connected via PE20 tubing, backfilled with saline with a small air bubble separating the saline from the peptide solution to a 10 μl Hamilton micro-syringe, driven by a microinjection pump (CMA/100, Carnegie Medicine; or PHD 2000, Harvard Apparatus, Holliston, MA, USA). The solution was injected at a rate of 0.5 μl per min. The total volume injected per amygdala was 0.5 μl. Pep-ephrinA4 peptide (Ac-RRQRYTPFPLGFE-Lys-(FITC)), GL Biochem, Shanghai, China) was dissolved in saline at a concentration of 10.4 μg/μl. Control inactive peptide is (Ac-RRWSGYEACTAEG-Lys(biotin)) was dissolved in saline at a concentration of 10.4 μg/μl. The control solution was saline with 35% DMSO (vol/vol). Following injection, the injection cannula was left for an additional 1 min before withdrawal to minimize dragging of injected liquid along the injection tract^20^.

### Acute systemic administration of the peptides

Rats were injected subcutaneously with pep-ephrinA4 or control peptide (0.2 mg of peptide in 0.25 ml saline)^20^.

### Fear conditioning and reconsolidation

Rats were habituated for 2 days to the training chamber (Coulbourn Instruments) for 30 min each day and briefly to the injection pump. On the next day, the animals were subjected to the fear conditioning protocol. Five minutes after the start of the training, animals were presented with a pairing of tone (conditioned stimulus (CS)—30 s, 5 kHz, 80 dB) that co-terminated with a foot shock (unconditioned stimulus (US)—0.5 s, 2 mA). Reactivation of memory was done 24 h after training by subjecting the animals to the CS tone in a different context (Formica floor no white light and infrared illumination). Three hundred seconds after the start of training the animal was subjected to a single tone (40 s, 5 kHz, 80 dB). Rat groups were tested in this different context 24 h after reactivation training for long-term memory. Three hundred seconds after the start of testing, animals were subjected to five-tone presentations (40 s, 5 kHz, 80 dB) with an inter-trial interval of 180 s on average. Behavior was recorded and the video images were transferred to a computer equipped with an analysis program (FreezFrama). The percentage of changed pixels between two adjacent 1-s images was used as a measure of activity.

### Histology

After the behavior was completed, the rats were decapitated and their brains were quickly removed, placed on dry ice and stored at − 80 °C until use. Brains were sliced (50 μm) and stained with cresyl violet acetate to verify cannula placements. Only rats with cannulas within the borders of the LA/BLA were included^20^. We excluded: in Fig. 1—2 rats from the peptide group and 1 from the control solution group; in Fig. 2—1 animal from the control solution group; in Fig. 3—1 rat from the peptide group and 2 animals from the control solution group; in Fig. 4—1 rat from the control inactive peptide group and 2 animals from the control solution group.

### Statistics

All experiments were statistically analyzed using SPSS. Behavioral analyses were performed using repeated measures ANOVA (for the long-term memory test with multiple tones) or t-test for the CS reactivation test (one tone). Differences were considered significant if p < 0.05. We have used the randomization within blocks approach. The outcome assessment and data analysis were done blindly.

## Data Availability

The datasets used and/or analyzed during the current study are available from the corresponding author on request.
